# Metabolic Diversity of Flavonoids and Antioxidant Potential for Characterization of Underutilized Citrus Species for Nutritional Security

**DOI:** 10.3390/plants11070862

**Published:** 2022-03-24

**Authors:** Dinesh Kumar, Milind Shivratan Ladaniya, Manju Gurjar, Sunil Kumar, Sachin Mendke

**Affiliations:** ICAR-Central Citrus Research Institute, Nagpur 440033, India; msladaniya@gmail.com (M.S.L.); manjugurjar966@gmail.com (M.G.); sunilut2004@gmail.com (S.K.); sachinmendke22@gmail.com (S.M.)

**Keywords:** hesperidin, naringin, ABTS, DPPH, FRAP, limonin, total phenol, dietary supplements

## Abstract

Citrus fruits are grown commercially throughout the world. They are widely consumed due to their nutrients, use in energy supplements, and numerous health benefits. There is significant interest among consumers about this naturally available source, rich in flavonoids and antioxidants. However, underutilized citrus varieties remain unexplored due to the lack of information about the pool of nutritive properties they confer. Ten underutilized citrus varieties were collected from Nokrek Biosphere Reserve, West Garo Hills, Meghalaya, India, identified by UNESCO as a Biosphere reserve, to study the diversity in terms of limonin, ascorbic acid, carotenoids, browning, flavonoids, total phenol, and antioxidant activity, the contents of which varied significantly among different citrus cultivars. The results indicated that Citron and Pomelo were good sources of ascorbic acid (29.50 and 45.09 mg/100 mL), and that Khasi papeda was found to contain lower limonin content (9.21 ppm). However, in terms of flavonoids, Khasi papeda and Pomelo were found to exhibit a higher naringin content (189.13 ppm and 32.15 ppm), whereas the hesperidin content was highest in Kachai lemon, Khasi papeda, and Chinotto, at 199.51 ppm, 148.04 ppm, and 135.88 ppm, respectively. Antioxidant activity was assessed by three antioxidant assays (ABTS^+^ (radical cation azino-bis [3-ethylbenzthiazoline-6-sulfonic acid]) (ABTS), 2,2-diphenyl-1-picrylhydrazyl radical (DPPH), and Ferric Reducing Antioxidant Power (FRAP)). Khasi papeda (7.48 mM L^−1^ Trolox), Chinotto (7.48 mM L^−1^ Trolox), and Pomelo (7.48 mM L^−1^ Trolox) exhibited the highest reducing power with DPPH radical scavenging activity, and Khasi papeda (15.41 mg GAE L^−1^) possessed a higher phenolic content, whereas the antioxidant activity when assessed with ABTS and FRAP assays was highest among the underutilized species of Khasi papeda (4.84 mM L^−1^ Trolox, 1.93 mM L^−1^ Trolox) and Ada Jamir (4.96 mM L^−1^ Trolox, 2.03 mM L^−1^ Trolox), respectively. To the best of our knowledge, this is among the very few papers presenting comprehensive data on the metabolic diversity of flavonoids and antioxidant potential to characterize the underutilized citrus species. This study also demonstrated that Khasi papeda, Pomelo, Chinotto, and Kachai lemon can serve as potential sources of functional components, bioactive compounds, and antioxidants, which can be explored for further application in the processing industry for nutritional security.

## 1. Introduction

Citrus fruits belong to the genus citrus and the family *Rutaceae* and are one of the most traded horticultural crops [1,2]. Citrus fruits have tremendous nutritional content and have potential demand in both the fresh and the processing markets [3]. These fruits are a treasure trove of phytochemicals and are an active source of functional components and bioactive compounds, including ascorbic acid, pectin, folic acid [4], flavonoids, carotenoids, total phenols, limonoids [2], and antioxidants [3]. The consumption of citrus fruits reduces the incidence and lowers the risk of several diseases such as cancers, cardiovascular and coronary heart disease, and stroke, and they exhibit other promising health benefits due to their anti-inflammatory, anti-tumor, and strong antioxidant activity [4,5,6].

The taxonomy of the genus citrus is not clear [1]. There are 16 and 144 species in the genus Citrus such as Swingle and Tanaka. Apart from the commercially available citrus fruits, viz. mandarin (*C. reticulata* Blanco), lime (*C. aurantifolia* Christm.), sweet orange (*C. sinensis* L. Osbeck), grapefruit (*C. paradisi* Osbeck), and lemon (*C. limon* L. Brn. F.) [7,8], India is bestowed with remarkable genetic diversity, including both wild and cultivated varieties [9] such as Indian wild orange (*C. indica* Tanaka), Kachai lemon (*C. jambhiri* Lush.), Ada jamir (*C. assamensis* Dutta and Bhattacharya), Chinotto (*C. myrtifolia* Raf.), Melanesian papeda (*C. macroptera* Montr.), Khasi papeda (*C. latipes* (Swingle) Tanaka), Citron (*C. medical* Linn.), Gajanimma (*C. pennivesiculata* (Lush.) Tanaka), Pomelo (*C. grandis* (L.) Osbeck or *C. maxima* (Burm.) Merr.), Galgal (*C. pseudolimon* Tanaka), karna or khatta orange (*C. karna* Raf.), *C. reshni*, *C. unshiu*, *Citrus* (*x paradisi*) *x trofilate*, *C. sinensis x poncirus trifoliate*, *C. depressa*, etc., which are grown in orchards or fruit gardens [6,10]. Different citrus types have originated due to mutations occurring spontaneously, natural selection, and hybridizations, thus leading to genetic modifications in the cultivars [1].

Although India is a reservoir and the natural home of various citrus species, these underutilized citrus fruits have not been explored on a commercial scale due to a lack of popularity and, at the same time, a lack of information among consumers about their nutritional value, in terms of bioactive compounds present and the antioxidant potential they exhibit [11]. Many underutilized wild citrus fruits that originated in north-east India prove to be predominant sources of carbohydrates, vitamins, minerals, and other phytochemicals [12], thus providing health benefits to humans. Systematic studies for the collection, characterization, and conservation of citrus genetic diversity are still far behind [10].

Looking to the dearth of information available and the importance of the varieties, an attempt was made to quantify the functional components, bioactive compounds, and antioxidant potential of wild and underutilized citrus species, which prove to be an alternative source to commercial citrus varieties in terms of nutritional security.

## 2. Results and Discussion

### 2.1. Limonin, Carotenoid and Total Phenol Content

Limonin is one of the oxygenated triterpenoids present in citrus juice and is mainly responsible for delayed bitterness. For the citrus juice industry, limonin is considered a major concern [13,14]. The limonin content varied from 9.21 ppm to 13.92 ppm, as given in Table 1.The limonin content was found to be significantly higher in Citron and was on par with Ada jamir. The underutilized varieties of Khasi papeda and Gajanimma showed the lowest values of 9.21 ppm and 10.01 ppm, respectively. On the other hand, Melanesian papeda (12.40 ppm), Ada jamir (13.41 ppm), and Citron (13.92 ppm) showed the highest limonin content. Limonin content was in the range of 6.82–32.40 ppm in different Pomelo cultivars [15]. Similar observations were also recorded [7] when carrying out a study with Kachai lemon, Citron, and Pomelo citrus varieties. Citrus varieties with low contents of limonin are mostly preferred in juice processing industries [7]. The limonin concentration varies with respect to species and is affected due to changing regional and cultural practices and nutrition [14].

In phytochemicals, carotenoid is one of the major contributors found in citrus juices [16]. For determining citrus juice quality, fat-soluble pigments are considered one of the principal parameters [17,18]. In the present study, carotenoid content revealed considerable diversity among the citrus species. The carotenoid content examined ranged from 0.10–0.86 mg/100 mL (Table 1). Even though most of the underutilized citrus varieties displayed a carotenoid content from 0.10–0.19 mg/100 mL, higher contents were obtained in three species, with Pomelo and Indian wild orange displaying potent antioxidant activity, i.e., the ability to scavenge reactive oxygen species harmful to the human body [19]. However, Chinotto and Citron contained almost identical amounts of carotenoid, and the rest of the species were found to be at par for carotenoid contents. Similar findings have previously been reported [7]. Various factors such as varietal, growing conditions, geographical origin, and the maturity of fruits influence the carotenoid content [18].

**Figure 1:**
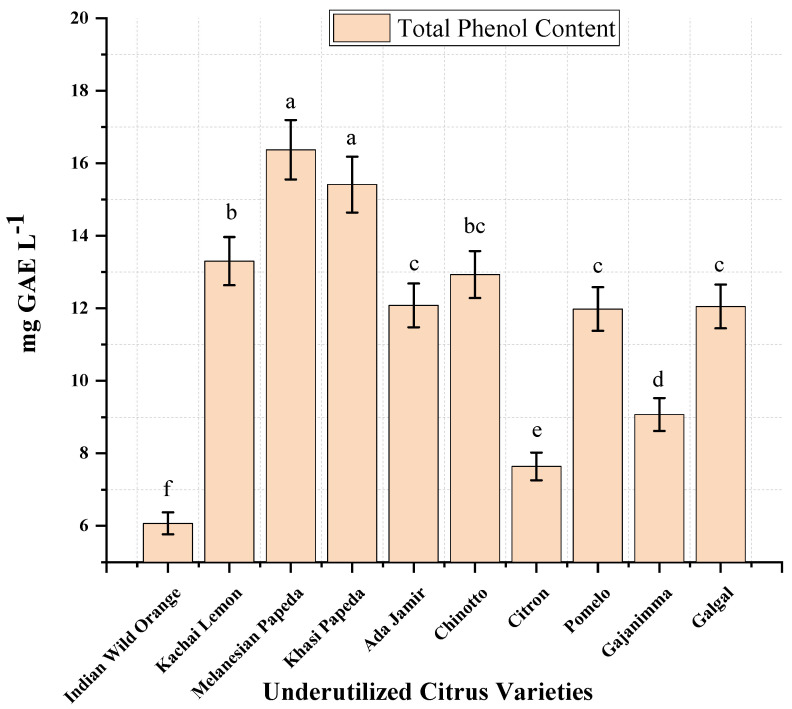
Total phenol compound content in underutilized citrus varieties. Significant ANOVA *p* < 0.01. Letters a to f above mean (n = 3) bars denote significant differences at *p* < 0.01 according to Tukey’s HSD test.

Total phenols are considered one of the major components depicting bioactive compound-enriched components [7]. The results of total phenols among underutilized citrus varieties are given in Figure 1 and are an average of three different replications. The total phenolic content varied from 6.07 mg GAE L^−1^ to 16.37 mg GAE L^−1^. Among the 10 different underutilized varieties studied, low TPC values were found in Indian wild orange (6.07 mg GAE L^−1^), Citron (7.64 mg GAE L^−1^), and Gajanimma (9.07 mg GAE L^−1^). Moderate values were also found in Pomelo, Galgal, Ada Jamir, and Chinotto (11.98–12.93 mg GAE L^−1^), whereas Kachai lemon, Khasi papeda, and Melanesian papeda contained relatively high amounts of phenols (13.30 mg GAE L^−1^, 15.41 mg GAE L^−1,^ and 16.37 mg GAE L^−1^, respectively). Phenolic compounds abundantly found in citrus fruits are known to possess significant antioxidant activity. The differences in the total phenol content observed in the results could be due to several environmental factors, namely climate, location, maturity period, temperature, rainfall, etc. [20,21]. The presence of different phenolic compounds might also be one of the causes for variation in the TPC observed [22].

### 2.2. Ascorbic Acid and Browning Content

Ascorbic acid is one of the major components to account for antioxidant activity in citrus species [16,23]. The determination of ascorbic acid content is of significance because the degradation of vitamin C leads to the formation of hydroxymethyl furfural (HMF) due to non-enzymatic browning, resulting in the browning of juice, considered unacceptable by consumers [24]. Variation in the amounts of ascorbic acid content is shown in Table 1. Pomelo (45.09 mg/100 mL) was found to contain the highest level of ascorbic acid. Chinotto (23.91 mg/100 mL), Kachai lemon (26.16 mg/100 mL), and Melanesian papeda (25.90 mg/100 mL) also presented high levels of ascorbic acid. The remaining underutilized citrus varieties had less ascorbic acid contents, which ranged from 8.11–25.09 mg/100 mL. The ascorbic acid content ranged from 6.80–37.92 mg/100 mL among the ten accessions of *C. indica* [25]. Similar results of ascorbic acid content in citron sp. were also reported when analyzing peel and pulp extracts, respectively [26]. Several researchers reported a wide variation in ascorbic acid (Vitamin C) content, not only in different citrus fruits but also in different cultivars within citrus species [27,28,29]. Several parameters such as climate and processing factors, cultural practices, and fruit quality, variety, and maturity, etc., are responsible for the variability in the ascorbic acid content [30,31,32].

The browning index content (also known as non-enzymatic browning (NEB)) is one of the most important chemical reactions responsible for citrus juice quality, determining color changes occurring due to the heat or storage of citrus juice products [33]. The browning index content in the underutilized citrus varieties examined in this study ranged from 0.10–0.52 O.D (Table 1). Significant variations were observed in the underutilized citrus varieties at *p* < 0.01. The highest browning index content was found in Melanesian papeda (0.52 O.D), followed by Ada jamir (0.29 O.D), Khasi papeda (0.23 O.D), and Kachai lemon (0.22 O.D), in order. Degradation of the ascorbic acid content is also one of the reasons for non-enzymatic browning. However, a reaction between sugars, amino acids, and ascorbic acid is also responsible for non-enzymatic browning [33].

### 2.3. Flavonoid Content

**Figure 2:**
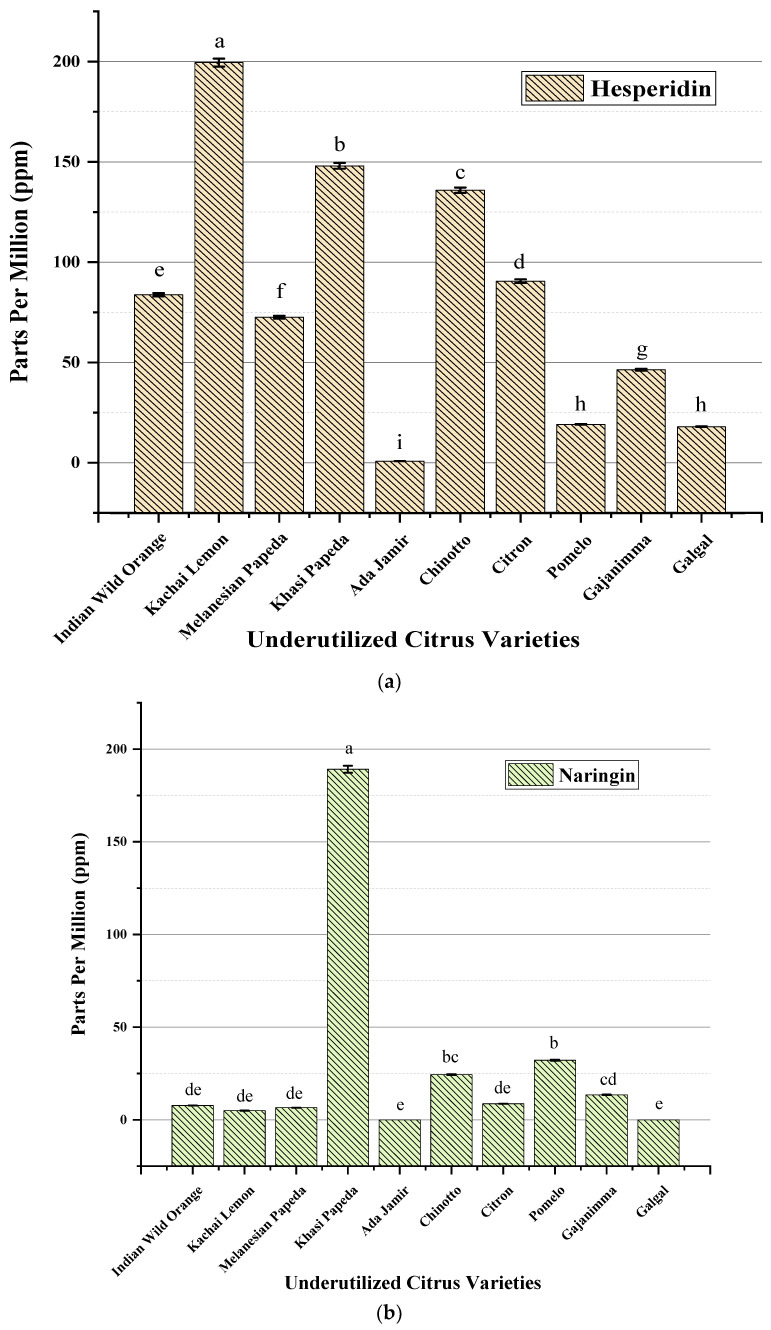
Flavonoid (**a**) hesperidin and (**b**) naringin content assessed in underutilized citrus varieties. Significant ANOVA *p* < 0.01. Letters a to i for hesperidin and a to e for naringin above mean (n = 3) bars denote significant differences at *p* < 0.01 according to Tukey’s HSD test.

Flavonoid constituents are found in substantial quantities in citrus species, some of which are characteristic of them [34]. Flavanone glycosides are specific to citrus and have been reported as stable bio-markers to differentiate citrus varieties [35]. The flavonoid contents, viz. hesperidin and naringin, in underutilized citrus species were quantified using HPLC, and the results are reported in Figure 2. The variation has been observed to be significantly higher in the species analyzed at *p* < 0.01. The highest flavonoid content (hesperidin 199.51 ppm) was found in Kachai lemon, Khasi papeda (hesperidin 148.04 ppm and naringin 189.13 ppm), and Chinotto (hesperidin 135.88 ppm and naringin 32.15 ppm). However, Ada jamir and Galgal showed the lowest hesperidin contents, with 0.79 ppm and 18 ppm, respectively. Further, naringin was not detected in these two underutilized varieties. Our findings indicated that among the flavanone glycosides tested, hesperidin was found in significant amounts in comparison to naringin. The results are in accordance with those of a previous study of different citrus varieties [36]. Genetic and physiological factors are the main causes for the variation in flavonoid content. In addition to these factors, the selection of a genotype with high ascorbic acid content is reported to influence the variation more than climatic conditions and cultural practices [37]. The obtained results indicated that three underutilized citrus species, namely Kachai lemon, Khasi papeda, and Chinotto, contained higher flavonoid contents in comparison to the other varieties assessed.

### 2.4. Antioxidant Activity

**Figure 3:**
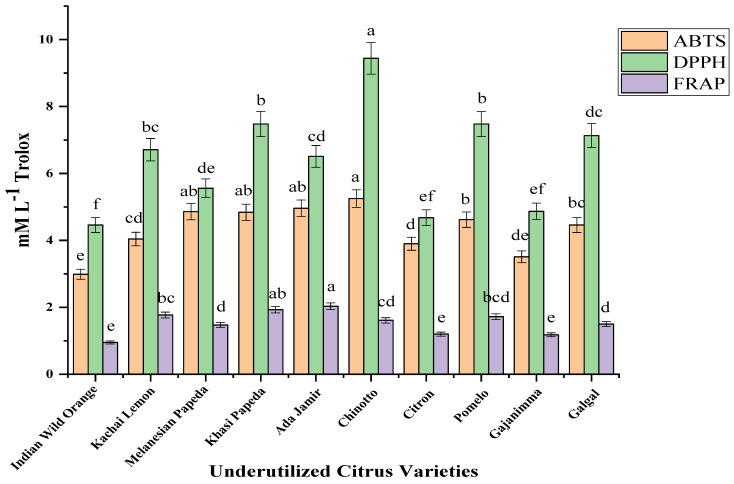
Antioxidant activity by ABTS, DPPH, and FRAP method in underutilized citrus varieties. Significant ANOVA *p* < 0.01. Letters a to f above mean (n = 3) bars denote significant differences at *p* < 0.01 according to Tukey’s HSD test.

In the present study, the in vitro antioxidant activity of underutilized citrus species was evaluated by combining more than one method, as different antioxidants present in food possess different mechanisms of action [24,38]. Therefore, the assays ABTS, DPPH, and FRAP, which are commonly accepted assays, are employed to investigate antioxidant activity. The ABTS, DPPH, and FRAP assays are based on an electron transfer, which involves the reduction of a color oxidant. The results of antioxidant assays using ABTS, DPPH, and FRAP scavenging are expressed in mM L^−1^ Trolox (depicted in Figure 3) and were found statistically significant at *p* < 0.01. In a specific consideration, the ABTS assay is based on the generation of a blue/green ABTS^+^ that can be reduced by antioxidants, whereas the DPPH assay is based on the reduction of the purple DPPH to 1,1-diphenyl-2-picryl hydrazine. The assays scavenge the free radicals of ABTS and DPPH, which leads to the formation of colorless products. The greater the degree of discoloration, the greater the scavenging of free radicals, and hence the greater the antioxidant activity of the samples [23]. The antioxidant activity measured by the ABTS method ranged from 2.99 to 5.25 mM L^−1^ Trolox. Concerning the DPPH assay, the values ranged from 4.46–9.44 mM L^−1^ Trolox. Among the studied varieties, Chinotto showed the highest antioxidant activity, whereas the lowest activity was observed in Indian wild orange when assessed with both the ABTS and DPPH assays. The ABTS assay is applicable to hydrophilic antioxidant systems, whereas the DPPH assay is applicable to lipophilic antioxidants. The FRAP assay is different from the others as there are no free radicals involved, but the reduction of ferric iron (Fe^3+^) to ferrous iron (Fe^2+^) is monitored. The FRAP assay measures the reduction capacity of the antioxidant present when reacting with a complex of ferric tripyridyltriazine (Fe^3+^-TPTZ) and leads to the formation of a colored ferrous tripyridyltriazine (Fe^2+^-TPTZ) complex [22]. The FRAP values in underutilized varieties varied from 0.95 mM L^−1^ Trolox (Indian wild orange) to 2.03 mM L^−1^ Trolox (Ada jamir). The results of our antioxidant activity tests showed that the varieties assessed can scavenge the oxygen reactive free radicals to a certain extent. The antioxidant activity measured may differ depending on the geographical origin, growing season, and agricultural practices.

## 3. Materials and Methods

### 3.1. Fruit Material

**Figure 4:**
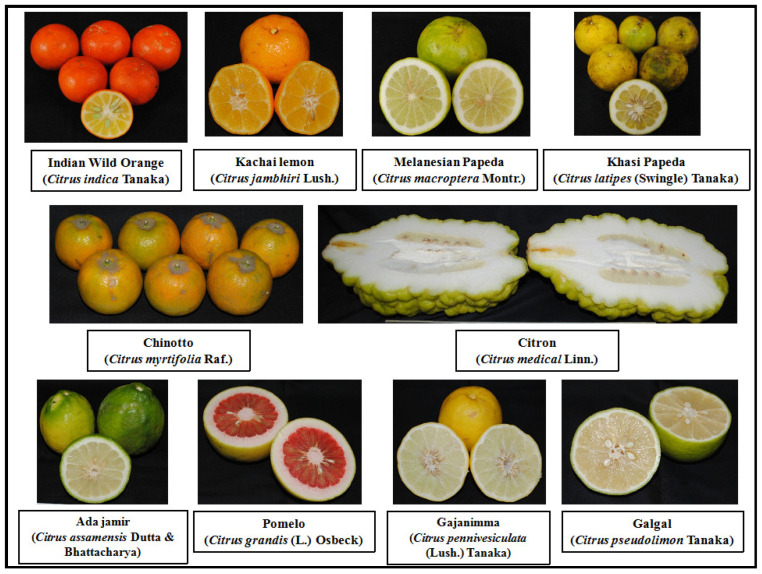
Underutilized citrus varieties collected from Nokrek Biosphere Reserve, West Garo Hills, Meghalaya, India.

Underutilized citrus fruits of different species, namely Indian wild orange (*Citrus indica* Tanaka), Kachai lemon (*Citrus jambhiri* Lush.), Melanesian papeda (*Citrus macroptera* Montr.), Khasi papeda (*Citrus latipes* (Swingle) Tanaka), Ada jamir (*Citrus assamensis* Dutta and Bhattacharya), Chinotto (*Citrus myrtifolia* Raf.), Citron (*Citrus medical* Linn.), Pomelo (*Citrus grandis* (L.) Osbeck or *Citrus maxima* (Burm.) Merr.), Gajanimma (*Citrus pennivesiculata* (Lush.) Tanaka), and Galgal (*Citrus pseudolimon* Tanaka), were harvested at maturity from Nokrek Biosphere Reserve, West Garo Hills, Meghalaya, India, identified by UNESCO as a Biosphere reserve (Figure 4). The fruits were washed in running water to remove the dust from the surface and then dried. The fruit juice was extracted after peeling and was stored in airtight containers until analyzed. The common and scientific names and probable place of origin of underutilized citrus varieties are given in Table 2.

### 3.2. Chemicals, Reagents and Standards

**Figure 5:**
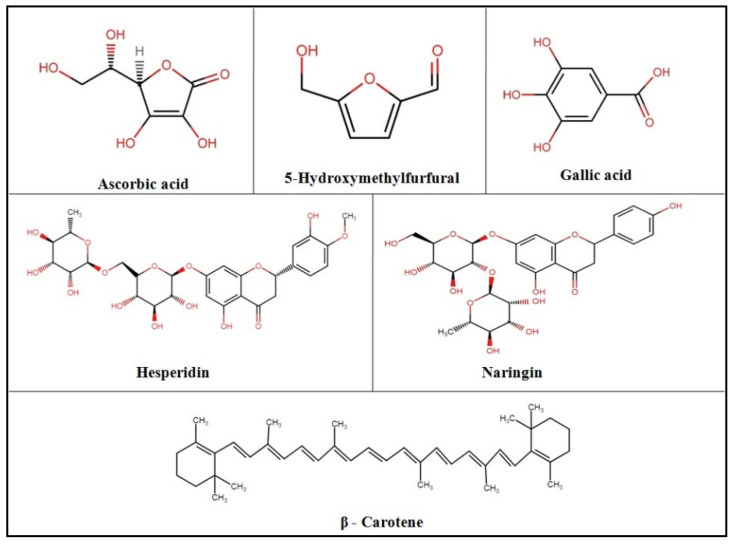
Chemical structure of ascorbic acid, 5-Hydroxymethylfurfural, gallic acid, hesperidin, naringin, and β-Carotene (ChemIDPlus database) [39].

The standards of ascorbic acid, limonin, carotenoids (β-carotene), flavonoids (hesperidin and naringin), Trolox, ABTS^+^ (radical cation azino-bis [3-ethylbenzthiazoline-6-sulfonic acid]), 2,2-diphenyl-1-picrylhydrazyl radical (DPPH), 2,4,6-Tri (2-pyridyl)-s-triazine (TPTZ), and total phenols (Gallic acid), and solvents such as water and acetonitrile of high-performance liquid chromatography (HPLC) grade were purchased from Sigma-Aldrich (Mumbai, India). All other chemicals and reagents used in the study were of analytical grade. Chemical structures of important compounds are depicted in Figure 5.

### 3.3. Physico-Chemical Analysis

#### 3.3.1. Ascorbic Acid Determination by HPLC

Ascorbic acid was determined using HPLC according to the method described [40,41]. An amount of 5 mL of 2.5% o-phosphoric acid was added to 5 mL of juice and centrifuged at 5000 rpm for 10 min. A 0.45 μm syringe filter (Millipore, Mumbai, India) was used for filtering supernatant and then injected into the HPLC system. A stationary phase C-18 column Zorbax SB C-18, 4.6 mm × 250 mm, 5 microns was used in combination with a mobile phase 20 mM Na_2_HPO_4_ at pH 2.5 for the elution of L-Ascorbic acid. The HPLC system was equipped with a quaternary pump DAD detector (M/s. Agilent Technologies, Bengaluru, Karnataka, India) (220 nm) with a flow rate of 1 mL/min. The ascorbic acid content was expressed as mg/100 mL of the juice sample.

#### 3.3.2. Browning Determination

Browning content was determined [42], and the results were expressed in optical density (O.D). An amount of 10 mL of the juice was centrifuged at 3000 rpm for 15 min. The juice supernatant was diluted with an equal amount of ethyl alcohol (95%) and centrifuged again at 10 min until a clear extract was obtained. The absorbance of the supernatant was read at 440 nm using a UV-spectrophotometer (UV-1650 PC Shimadzu, Kyoto, Japan).

### 3.4. Biochemical Analysis

#### 3.4.1. Limonin Determination

The limonin content was quantified [43] and was reported in parts per million (ppm). A total of 25 mL of juice was centrifuged at 3000 rpm for 10 min. An amount of 5 mL of supernatant was taken, and to this 10 mL of chloroform was added. Then, 4 mL of lower layer was taken out and 6 mL of freshly prepared Burncham reagent was added. The reaction mixture was kept at room temperature for 30 min, and the observation was recorded at 503 nm using a UV-spectrophotometer (UV-1650 PC Shimadzu, Kyoto, Japan).

#### 3.4.2. Carotenoid Determination

Carotenoid as β-carotene was determined according to the method [44]. Total carotenoid content was expressed as mg/100 mL of juice. An amount of 10 mL of the sample was mixed with 20 mL of methanol and 0.5 g celite and filtered with a Buchner funnel under vacuum pressure. The residue after filtration was blended with 20 mL of acetone in a mortar and pestle for 1 min for extraction of any colored particles. The extraction with acetone was repeated until the residue no longer contained any colored particles. The filtrate was collected in a separating funnel. An amount of 5 mL of n-hexane was mixed in and water was added until the layer separated. The aqueous acetone layer, i.e., the lower layer, was drained and discarded, and the hexane layer, i.e., the upper layer, was washed twice with water. Hexane solution was added to the colored hexane solution to make up 10 mL. n-hexane was used as a blank, and the absorbance was read at 450 nm using a UV-spectrophotometer (UV-1650 PC Shimadzu, Kyoto, Japan).

#### 3.4.3. Total Phenol Content Determination

The Folin-Ciocalteu assay [45] with some slight modifications was used for the determination of the total phenol content present in underutilized citrus varieties. In an Eppendorf microtube (1.5 mL), Milli-Q water, the sample, and the Folin-Ciocalteu reagent were added at a ratio of 790:10:50 μL and then vortexed. After exactly 1 min, 150 μL of 20% solution of sodium carbonate was mixed in, vortexed again, and allowed to stand for approximately 60 min under dark conditions. Absorbance was taken at 750 nm and quantified using a standard curve of gallic acid and reported as gallic acid equivalents (mg GAE L^−1^).

### 3.5. Flavonoids Determination using HPLC

For the quantification of flavonoids [46], samples were analyzed by a reverse phase HPLC system. An amount of 1 mL each of 15% (*w*/*v*) Carrez I (potassium hexacyanoferrate) and 30% (*w*/*v*) of Carrez II (zinc acetate dihydrate) solution was added into juice and centrifuged further at 6000 rpm for 10 min. The prepared samples of extracts and standards filtered through a 0.45 μm syringe filter (Millipore, Mumbai, India) were used for HPLC. A binary system (M/s. Agilent Technologies, Bengaluru, Karnataka, India) equipped with a PDA detector connected to a system processor was used for analysis. The HPLC solvents of 5mM ammonium acetate and acetonitrile (75:25 *v*/*v*) were run at a 284 nm wavelength using a reverse-phase C-18 column Zorbax SB C-18, 4.6 mm × 250 mm, 5 microns. During the run, a flow rate of 1mL/min was maintained using binary mode. In order to identify the compounds, standards of flavonoids (hesperidin, naringin) were used. The peaks were identified by comparing the retention time (RT) of the standard compounds with that of the different peaks obtained in the HPLC analysis of samples. Flavonoids, i.e., hesperidin and naringin contents, were expressed as mg/100 mL of juice sample.

### 3.6. Antioxidant Activity Determination by ABTS, DPPH, and FRAP Assay

To scavenge the ABTS^+^ radicals, the ABTS assay was adapted [24]. The absorbance was recorded at 414 nm. Results were expressed as mmol L^−1^ Trolox.

The DPPH activity was estimated [24]. The absorbance of the reaction mixture was read at 515 nm. The results were expressed as mmol L^−1^ Trolox.

The procedure [47] with minor modifications was performed for the Ferric Reducing Antioxidant Power (FRAP) assay as a measure of antioxidant power. In this method, the antioxidants in the samples are reduced to blue ferrous form, which has absorption maxima at 593 nm. The working reagent of FRAP was prepared freshly by adding 300 mM of acetate buffer, TPTZ solution (0.3123%), and ferric chloride (FeCl_3_) (0.54%) solutions at a ratio of 10:1:1. An amount of 2 μL diluted extract of the sample and 250 μL of the FRAP reagent were added to each well. The absorbance of samples was recorded at 593 nm. The Trolox standard was used for the quantification and preparation of the calibration curve. The results were expressed in mmol L^−1^ Trolox.

### 3.7. Statistical Analysis

The results obtained are the means of three replicated trials. The values are given in means ± standard deviation for verifying the statistical significance among the parameters. A multiple range test (Tukey’s HSD test) and an analysis of variance (ANOVA) were carried out to determine the significance of the test. The probability values of the laboratory experiments, which are usually calculated with (*p* < 0.01), were adopted as statistically significant.

## 4. Conclusions

The study of the diversity of phytochemical profiling and antioxidants among underutilized citrus species revealed significant diversity. The underutilized varieties, namely Khasi papeda, Pomelo, Chinotto, and Kachai lemon, have tremendous potential in the citrus industry because these varieties exhibit a range of functional components and bioactive compounds with antioxidant capacity. These citrus varieties are underutilized and unexplored, besides being a natural supply of phytochemicals and antioxidants. The antioxidant assays of ABTS, DPPH, and FRAP can be considered useful tools for determining antioxidant activity. The results of the study showed that the underutilized citrus species can be regarded as potential resources that are unexplored and capable of offering dietary supplements to the processing industry, as they are rich in nutrients at a significantly lower cost. Future studies can also be undertaken by other researchers to characterize the exotic and wild citrus germplasm from native locations, and to determine their promise in the food industry by providing natural sources and real benefits of inherent nutrients.

## Figures and Tables

**Table 1:** Ascorbic acid, carotenoid, and browning content quantified in underutilized citrus varieties collected from Nokrek Biosphere Reserve, West Garo Hills, Meghalaya, India.

Sr. No.	Varieties	Ascorbic Acid (mg/100 mL)	Carotenoid (mg/100 mL)	Limonin (ppm)	Browning (O.D)
1	Indian wild orange	13.23 ^g^ ± 0.29	0.86 ^a^ ± 0.05	11.37 ^cd^ ± 0.32	0.17 ^cde^ ± 0.02
2	Kachai lemon	26.16 ^c^ ± 1.37	0.29 ^b^ ± 0.08	11.76 ^cd^ ± 0.50	0.22 ^bc^ ± 0.03
3	Melanesian papeda	25.90 ^cd^ ± 0.77	0.15 ^c^ ± 0.01	12.40 ^bc^ ± 0.25	0.52 ^a^ ± 0.03
4	Khasi papeda	21.87 ^ef^ ± 1.01	0.12 ^c^ ± 0.02	9.21 ^f^ ± 0.10	0.23 ^bc^ ± 0.03
5	Ada jamir	22.88 ^de^ ± 0.48	0.15 ^c^ ± 0.02	13.41 ^ab^ ± 0.28	0.29 ^b^ ± 0.02
6	Chinotto	23.91 ^cde^ ± 0.79	0.19 ^bc^ ± 0.01	11.63 ^cd^ ± 0.14	0.10 ^e^ ± 0.04
7	Citron	29.50 ^b^ ± 1.72	0.19 ^bc^ ± 0.04	13.92 ^a^ ± 0.19	0.18 ^cde^ ± 0.03
8	Pomelo	45.09 ^a^ ± 0.38	0.75 ^a^ ± 0.05	10.72 ^de^ ± 0.42	0.19 ^cd^ ± 0.02
9	Gajanimma	8.11 ^h^ ± 0.29	0.10 ^c^ ± 0.02	10.01 ^ef^ ± 0.48	0.14 ^cde^ ± 0.01
10	Galgal	19.58 ^f^ ± 0.83	0.11 ^c^ ± 0.01	11.61 ^cd^ ± 0.25	0.12 ^de^ ± 0.01
Tukeys HSD at 1%		3.2067	0.1253	1.1236	0.0875

The given values are in mean ± standard deviation (n = 3). According to Tukey HSD multiple range test, means with superscripts in the columns followed by different letters are significantly different at *p* < 0.01. Means with superscripts in each column with the same letter do not differ significantly at *p* < 0.01. Superscript letters denote significant differences at *p* < 0.01 according to Tukey’s HSD test.

**Table 2:** Underutilized citrus varieties (common name, scientific name, and probable place of origin).

Sr. No.	Common Name	Scientific Name	Centre of Origin
1	Indian wild orange	*Citrus indica* Tanaka	Northeast India
2	Kachai lemon	*Citrus jambhiri* Lush.	Northeast India
3	Melanesian papeda	*Citrus macroptera* Montr.	Southeast Asia
4	Khasi papeda	*Citrus latipes* (Swingle) Tanaka	Northeast India
5	Ada jamir	*Citrus assamensis* Dutta and Bhattacharya	Northeast India
6	Chinotto	*Citrus myrtifolia* Raf.	Southern China
7	Citron	*Citrus medical* Linn.	India
8	Pomelo	*Citrus grandis* (L.) Osbeck or *Citrus maxima* (Burm.) Merr.	Polynesia and Malay
9	Gajanimma	*Citrus pennivesiculata* (Lush.) Tanaka	South India
10	Galgal	*Citrus pseudolimon* Tanaka	India

## Data Availability

Samples of the data are provided by the corresponding author on request.
